# Identification of genes and miRNA associated with idiopathic recurrent pregnancy loss: an exploratory data mining study

**DOI:** 10.1186/s12920-020-00730-z

**Published:** 2020-06-01

**Authors:** Wael Bahia, Ismael Soltani, Anouar Abidi, Anis Haddad, Salima Ferchichi, Samia Menif, Wassim Y. Almawi

**Affiliations:** 1 Research Unit of Clinical and Molecular Biology, Department of Biochemistry, Faculty of Pharmacy of Monastir, University of Monastir, Monastir, Tunisia; 2 Faculty of Science of Bizerte, University of Carthage, Bizerte, Tunisia; 3 Molecular and Cellular Hematology Laboratory, Institut Pasteur de Tunis, Université Tunis El Manar, Tunis, Tunisia; 4 Laboratory of Physiology, Faculty of Medicine of Tunis, la Rabta, 1007 Tunis, Tunisia; 5 Department of Obstetrics and Gynecology, Fattouma Bourguiba University Hospital, Monastir, Tunisia; 6 Faculty of Sciences, El Manar University, Tunis, Tunisia; 7 College of Health Sciences, Abu Dhabi University, Abu Dhabi, United Arab Emirates

**Keywords:** Bioinformatics analysis, Gene expression, microRNA, Recurrent pregnancy loss

## Abstract

**Background:**

Recurrent pregnancy loss (RPL) is a significant adverse pregnancy complication, with an incompletely understood pathology. While many entities were proposed to elucidate the pathogenic basis of RPL, only few were significant enough to warrant investigation in all affected couples.. The aim of this study was to provide novel insights into the biological characteristics and related pathways of differentially expressed miRNA (DEMs) and genes (DEGs), in RPL, and construct a molecular miRNAs–mRNAs network.

**Methods:**

miRNAs and gene expression data were collected, and a number of DEMs and (DEGs) were obtained, and regulatory co-expression network were constructed. Function and enrichment analyses of DEMs were conducted using DIANA-miRPath. DEGs were screened, and were used in generation of protein-protein interaction (PPI) network, using STRING online database. Modularity analysis, and pathway identification operations were used in identifying graph clusters and associated pathways. DEGs were also used for further gene ontology (GO) analysis, followed by analysis of KEGG pathway.

**Results:**

A total of 34 DEMs were identified, and were found to be highly enriched in TGF-β signaling pathway, Fatty acid metabolism and TNF signaling pathway. Hub miRNAs were selected and were found to be involved in several functional pathways including progesterone-mediated oocyte maturation and Thyroid hormone signaling pathway. Five dysregulated feedback loops involving miRNA and TFs were identified and characterized. Most notably, PPI network analysis identified hub-bottleneck protein panel. These appear to offer potential candidate biomarker pattern for RPL diagnosis and treatment.

**Conclusions:**

The present study provides novel insights into the molecular mechanisms underlying RPL.

## Background

Recurrent pregnancy loss (RPL), defined as two or more consecutive pregnancy losses before the 20th week of gestation, is a significant pregnancy complication, affecting 2–5% of pregnancies [1]. While its exact etiology remains poorly understood [2] several risk factors were shown to influence the risk of RPL These include endocrine, immunologic, infectious, and genetic factors [3]. Whereas increased RPL susceptibility was linked with carriage of select gene variants, few of these at-risk variants was confirmed to contribute to RPL pathogenesis.

MicroRNAs (miRNA) are short (22–23 nucleotides) non-coding RNA, which regulate post-transcriptional activities [4], reportedly regulating ~ 60% of all protein-coding human genes, and are involved in diverse physiological processes and pathological states [5]. Their activity is attributed to their gene silencing capacity, by which miRNA binds > 100 target mRNA sites with partial base complementarity, thus preventing de novo translation, and/or accelerating mRNA degradation. Collectively, this constitutes an important regulatory feature of the transcriptome [6]. miRNAs are co-expressed with their host genes [7], and thus influence downstream signaling events [8]. Dysregulated miRNA expression was associated with physiological and pathological processes, including cellular differentiation, angiogenesis, apoptosis, and embryogenesis [9, 10].

There is growing interest in the contribution of gene-environmental interaction in RPL, and miRNA were proposed to constitute RPL at-risk factors [11, 12]. Given their diversity in regulating gene expression, the exact scope of action and influence of co-regulators and associated factors on miRNA activity remained poorly understood. Network modelling was proposed as key for deciphering miRNA activity. While this was described for number of pathological processes, miRNA network analysis in RPL pathogenesis was not previously reported.

The aims of the current study were to integrate the independent and different datasets in the analysis, thus overcoming the limitations caused by sample size and bias of statistical method. By summarizing large-scale gene expression data into limited modules, the study also aims to simplify data complexity, thus clarifying systematically the pathogenic mechanisms underlying RPL. Using direct association rationale, we provide for organization or connection information within module miRNA-genes. This may underscore the contribution of unannotated miRNA and genes to RPL pathogenesis.

## Methods

### Data collection and preprocessing

In the present study, the differentially expressed genes (DEGs) included genetically mutated genes, abnormally expressed protein genes, copy number variants, genes that alter DNA methylation, single nucleotide polymorphisms (SNPs), downregulated and upregulated genes. DEGs in the RPL were extracted from multiple databases (Additional file 1: Table S1). These databases include Online Mendelian Inheritance in Man (OMIM: catalog of human genes and genetic disorders that is freely available and updated daily) [13], GeneCards [14], Orphanet [15], Genetic Association Database (GAD, database of genetic association data from complex diseases and disorders) [16], and HuGE Navigator (provides a disease-centered view of genetic association studies and information about genes explored in relation to a unique disease) [17]. We conducted a systematic review aimed at identifying genes with differential expression in RPL patients and tissues, using a combination of the key terms recurrent pregnancy loss, habitual abortion, idiopathic RPL, spontaneous recurrent miscarriage, associated polymorphisms, gene mutations, and gene expression profiling.

A list of genes associated with RPL included genes associated with cell adhesion (trophoblast/endometrium interaction), dysregulated immunity, coagulation and angiogenesis, and cell survival were also collected from literature search (Additional file 1: Table S1). P-match tool which is closely interconnected with the TRANSFAC® database, was utilized in identifying DNA transcription factor binding sites (TFBS) [18]. We focused only on TFs obtained using TransmiR. Prior to P-match, 1000 nt promoter sequences of differentially expressed miRNAs were downloaded from Ensembl [19], using Regulatory Sequence Analysis Tools (RSAT) [20]. P-match matrix library comprises known TFBSs extracted from TRANSFAC [21], which allows searching of distinct TFBS. The “high quality vertebrate matrices only” option was selected as default, to reduce false-positive validation using P-match.

### Differentially expressed miRNAs in RPL

Differentially expressed miRNA (DEM) linked with RPL were extracted from miR2Disease [22], PhenomiR [23], and the Human microRNA Disease Database (HMDD) [24], using the following keywords: recurrent pregnancy loss, idiopathic RPL, spontaneous recurrent miscarriage AND microRNA (Additional file 1: Table S1). Additionally, we performed a secondary research based on systematic literature review of articles published between 2010 and 2017, and re-analyzed experimentally validated human miRNAs expression signatures in RPL from various biological sources (peripheral blood, and cell lines e.g., extravillous cytotrophoblast-derived cell line HTR8/SVneo (HTR8)) and information on aberrantly regulated miRNAs in patients with RPL as compared to healthy individuals. Our total search revealed 72 DEMs in RPL, as compared to healthy controls. Conflicting results have been found regarding expression levels of miRNAs. In order to increase the accuracy of our findings, only 37 of the most frequent DEM were collected (Table 1).

**Table 1:** Differentially expressed microRNAs list

Name	State	Sequence	Fold change	*P*-Value
hsa-miR-146a-5p	UP	UGAGAACUGAAUUCCAUGGGUU	4.45436783	0.0009
hsa-miR-125a-5p	UP	UCCCUGAGACCCUUUAACCUGUGA	2.79	< 0.001
hsa-miR-155-5p	UP	UUAAUGCUAAUCGUGAUAGGGGUU	3.96	< 0.001
hsa-miR-221-3p	UP	AGCUACAUUGUCUGCUGGGUUUC	7.409	0.0070
hsa-miR-146b-5p	UP	UGAGAACUGAAUUCCAUAGGCUG	5.108	0.0087
hsa-miR-320b	UP	AAAAGCUGGGUUGAGAGGGCAA	2.637	0.0022
hsa-miR-133a-3p	UP	UUUGGUCCCCUUCAACCAGCUG	0.009684	< 0.001
hsa-miR-101-3p	UP	UACAGUACUGUGAUAACUGAA	3.614	0.0193
hsa-miR-223-3p	UP	UGUCAGUUUGUCAAAUACCCCA	5.7	< 0.001
hsa-miR-92a-3p	UP	UAUUGCACUUGUCCCGGCCUGU	2.662	0.0308
hsa-miR-148b-3p	UP	UCAGUGCAUCACAGAACUUUGU	4.595	0.0434
hsa-miR-30d-5p	UP	UGUAAACAUCCCCGACUGGAAG	4.361	0.0285
hsa-miR-23b-3p	UP	AUCACAUUGCCAGGGAUUACCAC	3.430	0.0210
hsa-miR-423-3p	UP	AGCUCGGUCUGAGGCCCCUCAGU	2.01	0.0332
hsa-miR-145-5p	UP	GUCCAGUUUUCCCAGGAAUCCCU	0.722968	0.5116
hsa-miR-16-5p	UP	UAGCAGCACGUAAAUAUUGGCG	0.879078	0.3559
hsa-miR-181a-5p	UP	AACAUUCAACGCUGUCGGUGAGU	2.854288	0.0925
hsa-miR-424-5p	UP	CAGCAGCAAUUCAUGUUUUGAA	0.127032	0.8565
hsa-miR-30d-5p	UP	UGUAAACAUCCCCGACUGGAAG	0.604136	0.1879
hsa-miR-143-3p	UP	UGAGAUGAAGCACUGUAGCUC	1.336424	0.2162
hsa-miR-27b-3p	UP	UUCACAGUGGCUAAGUUCUGC	1.052276	0.4425
hsa-miR-17-5p	Down	CAAAGUGCUUACAGUGCAGGUAG	0.35	0.0020
hsa-miR-19b-3p	Down	UGUGCAAAUCCAUGCAAAACUGA	0.34	< 0.001
hsa-miR-559	Down	UAAAGUAAAUAUGCACCAAAA	0.390	0.0150
hsa-miR-365a-3p	Down	UAAUGCCCCUAAAAAUCCUUAU	0.321	0.0318
hsa-miR-1182	Down	GAGGGUCUUGGGAGGGAUGUGAC	0.238	0.0186
hsa-miR-4284	Down	GGGCUCACAUCACCCCAU	0.428	0.0079
hsa-miR-4264	Down	ACUCAGUCAUGGUCAUU	0.113	0.0013
hsa-miR-4474-5p	Down	UUAGUCUCAUGAUCAGACACA	0.196	0.0399
hsa-miR-22-5p	Down	AGUUCUUCAGUGGCAAGCUUUA	0.337	0.0172
hsa-miR-372-5p	Down	CCUCAAAUGUGGAGCACUAUUCU	0.22803727	0.0021
hsa-miR-451a	Down	AAACCGUUACCAUUACUGAGUU	−1.31401	0.0592
hsa-miR-21-5p	Down	UAGCUUAUCAGACUGAUGUUGA	−1.45161	0.4086
hsa-miR-149-5p	Down	UCUGGCUCCGUGUCUUCACUCCC	−1.530395	0.1853
hsa-miR-181b-5p	Down	AACAUUCAUUGCUGUCGGUGGGU	−2.329242	0.0434

### Experimentally validated miRNA associations

Experimentally validated human miRNAs and miRNA-target interaction (MTI) datasets were extracted from miRtarbase and miRecords [25], and were validated by reporter assays, Western blotting, or microarrays with miRNA over-expression or knockdown [26]. Individual TFs were mapped to human TF list in ChIPBase, to distinguish among target genes [27]. Experimentally-confirmed TF-miRNAs regulations were extracted from TransmiR for assessing TF-miRNAs interplay [28]. This included information about miRNAs function, disease associations, and type of regulation (activation/repression). This association mapping between miRNAs and their host genes was done using NCBI, and miRBase, the latter containing information on 38,589 mRNA by March 2018 [29].

### Construction of differentially expressed and transcriptional networks

A global network was constructed using CyTargetlinker [30], following extraction of regulatory associations between TFs, miRNAs, target genes and host genes. BridgeDb identifier mapping framework [31] was employed in standardizing miRNAs and genes designation, while Cytoscape was used in constructing differentially expressed networks (Additional file 1: Table S1). Lines between differentially-expressed nodal factors signify the interaction between different TFs, genes, and miRNAs in RPL; single nodes lacking regulatory miRNA association wereexcluded.

### Protein–protein interaction (PPI) network

Protein–protein interaction (PPI) network, which identifies key elements in RPL based on interaction level, was acquired from STRING (Search Tool for Retrieval of Interacting Genes) database. DEG were mapped to STRING to evaluate PPI information, and visualized with Cytoscape. In addition, clusters of network highly intra-connected nodes were searched by MCODE (Molecular Complex Detection) Cytoscape network plug-in (Additional file 1: Table S1) [32].

### Functional enrichment analysis

Gene ontology (GO) functional enrichment analysis was done using BiNGO (Biological Networks Gene Ontology) [33]. *P* values were correct for false discovery rate (FDR) using Benjamini-Hochberg multiple testing correction. Kyoto Encyclopedia of Genes and Genomes (KEGG) pathway enrichment analysis of differentially expressed miRNA was performed by DNA Intelligent Analysis (DIANA)-miRPath v2.0 (Additional file 1: Table S1). Following DEMs inclusion miRNAs targets predicted with DIANA-microT-CDS and/or experimentally validated transcripts from TarBase v6.0 were selected; *P-*value for each pathway was obtained by Fisher’s method [34]. The combinatorial effect of co-expressed miRNAs was evaluated by simultaneous analysis of multiple miRNAs; default settings were score cutoff of 0.8 for target prediction of 350 mRNA targets per miRNA, FDR to correct multiple hypothesis testing, and *P*-value of 0.05.

## Results

The occurrence and development of RPL are a complex process that involves genetic and epigenetic disorders. In the current study, a system-based networks’ approach was used to examine the key regulatory associations between miRNAs, their target genes, their host genes, and TFs in RPL process. Experimentally validated data from databases and literature was collected and networks were constructed. Due to their complexity, data associated with this network does not provide for a clear description of the findings. Accordingly, we focused on analysis of differentially expressed network, RPL transcriptional network, and PPI network.

### Differentially expressed miRNA and gene network of RPL

**Fig. 1:**
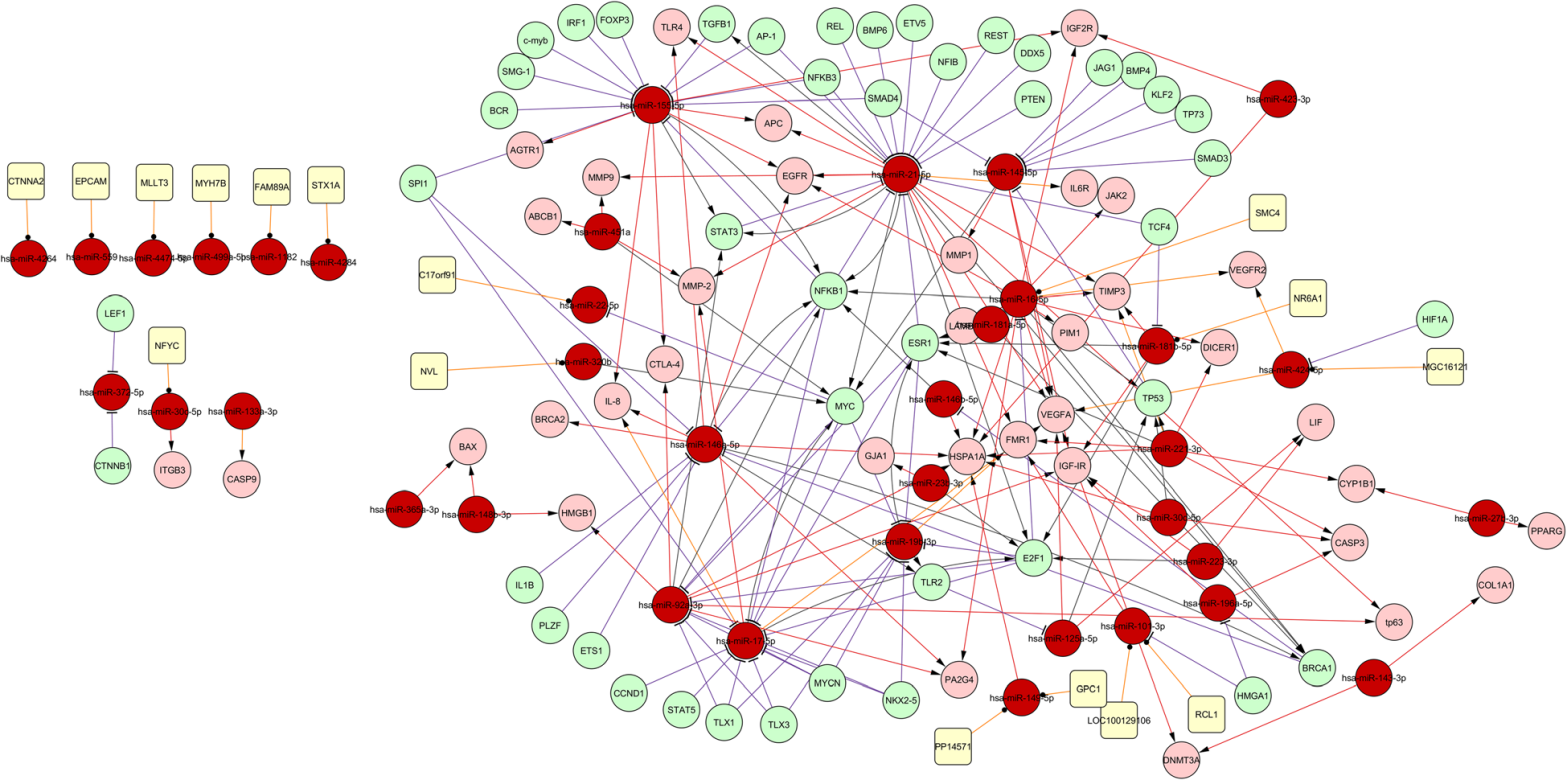
Differentially expressed network in RPL. Symbols used and their indications were: miRNA (), T (), Target gene (), host gene (), miRNA host gene (), transcriptional regulation (), and miRNA targeting ()

A regulatory network for up- and down-regulated miRNAs and genes, was constructed for interpreting their differential expression in RPL at the transcriptional level (Fig. 1). This network, comprising 133 nodes/206 edges, consisted of 44 TFs, 36 target genes, 37 miRNAs, and associated host genes (Additional file 2: Table S2). Except for host genes, all other nodes were differentially expressed. The network nodes, directly or indirectly, affect other nodes. When combined, the interactions reveal the primary cause of RPL. The unaltered genes and miRNAs suggest that they are not related to RPL, they might not be actually protective. This suggests that RPL may be prevented by targeting these differentially expressed elements. miRNAs with high connectivity and module membership within a module were defined as “hub miRNAs”, and were assumed to be functionally relevant. The top-10 hub miRNAs with the highest connectivity and module membership, were (in order): hsa-miR-21-5p, hsa-miR-155-5p, hsa-miR-16-5p, hsa-miR-17-5p, hsa-miR-146a-5p, hsa-miR-92a-3p, hsa-miR-145-5p, hsa-miR-19b-3p, hsa-miR-221-3p and hsa-miR-101-3p (Additional file 2: Table S2). These were selected based on the module sizes as hub miRNAs for further study.

As individual miRNA regulates gene expression, and multiple miRNAs modulate specific pathways, we explored the pathways regulated by hub miRNAs in RPL. Following inclusion of upregulated miRNAs, DIANA-miRPath identified 60 pathways as significantly enriched (*P* < 0.05). For simplicity and specificity, we focused only on pathways linked to RPL, the top 30 of which are shown in Table 2 (Additional file 2: Table S2). The most significantly enriched pathways regulated by hub miRNAs were TGF-β signaling, cell cycle, adherens junction, fatty acid elongation, progesterone-mediated oocyte maturation, thyroid hormone signaling, and MAPK signaling pathways, which in turn target NF-κB1, MYC and E2F1 hub genes. Interestingly, the estrogen signaling pathway, which modulates reproductive functions, including progesterone production, utero placental blood flow, female secondary sexual characteristics, and maintenance of pregnancy, is also regulated by the selected hub miRNAs. Alteration in this pathway represents a cause of implantation failure and recurrent miscarriage. Significant enrichment was seen in 15 pathways influenced by hsa-miR-21-5p, hsa-miR-17-5p, and hsa-miR-19b-3p (Table 2).

**Table 2:** Top 15 pathways significantly influenced by upregulated and downregulared miRNAs in RPL

KEGG pathway	***p***-value	Genes	miRNAs
Upregulated miRNAs			
TGF-beta signaling pathway	3.89 × 10^−11^	52	7
Adherens junction	1.93 × 10^−10^	51	7
Cell cycle	5.00 × 10^−8^	78	7
p53 signaling pathway	4.11 × 10^−5^	44	7
FoxO signaling pathway	5.05 × 10^−5^	73	7
Protein processing in endoplasmic reticulum	5.05 × 10^−5^	88	7
Fatty acid biosynthesis	1.38 × 10^−4^	4	4
Oocyte meiosis	1.85 × 10^−4^	58	7
Fatty acid metabolism	3.93 × 10^−4^	21	5
TNF signaling pathway	7.64 × 10^−4^	60	7
Estrogen signaling pathway	6.65 × 10^−3^	49	7
NOD-like receptor signaling pathway	0.018	30	7
HIF-1 signaling pathway	0.030	51	7
MAPK signaling pathway	0.040	111	7
Circadian rhythm	0.042	18	7
Downregulared miRNAs			
Thyroid hormone signaling pathway	9.24 × 10^−7^	50	3
ECM-receptor interaction	3.72 × 10^−6^	24	3
Fatty acid elongation	8.50 × 10^−5^	9	3
Signaling pathways regulating pluripotency of stem cells	1.24 × 10^−4^	50	3
Prolactin signaling pathway	1.96 × 10^− 4^	32	3
mRNA surveillance pathway	3.98 × 10^−4^	37	3
Lysine degradation	7.17 × 10^−4^	18	3
Insulin signaling pathway	1.80 × 10^−3^	52	3
Progesterone-mediated oocyte maturation	2.40 × 10^− 3^	36	3
Apoptosis	2.80 × 10^−3^	32	3
Ubiquitin mediated proteolysis	3.45 × 10^−3^	52	3
PI3K-Akt signaling pathway	4.35 × 10^−3^	102	3
RNA degradation	6.25 × 10^−3^	31	3
RNA transport	0.012	54	3
Endocytosis	0.033	67	3

### Proposed transcriptional network of predicted TFs

**Fig. 2:**
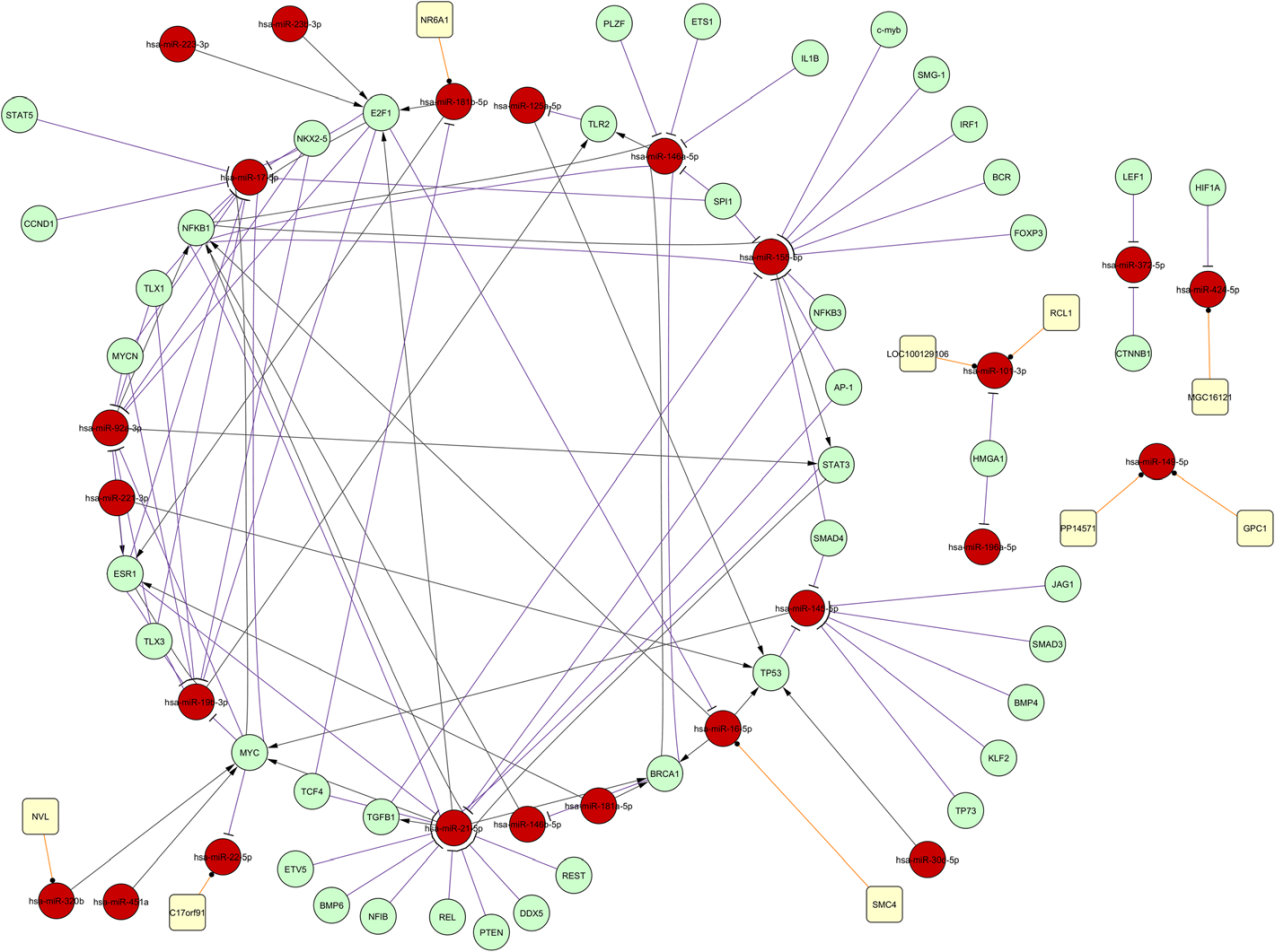
The Transcriptionnal Network in RPL. Symbols used and their indications were: miRNA (), TF () host gene (), miRNA host gene (), transcriptional regulation (), and miRNA targeting ()

We predicted that DEM may contribute to targeting TF, since differentially expressed genes affect nucleic acid binding, and thus TF protein-binding activities. This allows assessment of the regulatory relationship between DEM and key TF genes. Analysis with predicted (P-match) and validated (transmiR) databases provided for construction of complex network, in which motifs between DEM and popular TFs were the building blocks (Fig. 2). A total of 44 TFs co-regulate 37 DEMs. Most proteins in this network are TFs that affect diverse cellular processes, such as angiogenesis, DNA damage response, development, morphogenesis, differentiation, and survival. Deregulated feedback in STAT3, NF-κB1, ESR1, MYC and E2F1 5 loops were also identified in this network.

Targeting NF-κB1 by hsa-miR-92a-3p, hsa-miR-16-5p, hsa-miR-155-5p, hsa-miR-146a-5p, hsa-miR-21-5p, and hsa-miR-146b-5p, regulating hsa-miR-21-5p, hsa-miR-155-5p, hsa-miR-146a-5p, and hsa-miR-17-5p expression. NF-κB1 is involved in two distinct apparently counter-regulatory feedback loops. The first involves NF-κB1 alone, and the second involving hsa-miR-155-5p and hsa-miR-146a-5p. Heightened expression of hsa-miR-155-5p decreases *NF-κB1* gene expression, resulting in reduced hsa-miR-155-5p expression. This increases *NF-κB1* and subsequently hsa-miR-155-5p expression, and reactivates the loop. Similarly, feedback loop operates for *NF-κB1*/hsa-miR-146a-5, which oscillates between increased and decreased states. This suggests that mixed regulation existing between hsa-miR-155-5p and hsa-miR-146a-5 with NF-κB1 involves multiple targets and pathways contributing to RPL. NF-κB1 is also involved in a double negative feedback loop with hsa-miR-21-5p, allowing for “switch” type behavior, in which either NFKB1 or hsa-miR-21-5p, but not both, is expressed. The transition from one expression state to another state in this loop represents external modification of NF-κB1 or hsa-miR-21-5p expression.

On the other hand, hsa- miR-21-5p forms a separate feedback loop in targeting its regulators, NF-κB1 and STAT3. IntAct database and our PPI network confirm direct binding of NF-κB1 to several TFs, including STAT3, p53, ESR1, ATF3, SMAD3 and SMAD4. This suggests the existence of forward loop between ESR1, NF-κB1 and has-miR-21-5p, which precipitates a deregulated mechanism. This three-gene pattern comprises an inducer (ESR1), which regulates the transcription of hsa-miR-21-5p, and both jointly regulating NF-KB1 activation. By targeting the same genes, the predicted network demonstrates that cross TF cooperation is needed for miRNA expression, highlighted by the regulation of hsa-miR-17-5p by STAT5, hsa-miR-21-5p by STAT3, and both hsa-miR-17-5p and hsa-miR-21-5p targeting MMP-2 expression.

Specific TF can cooperate with other TF in regulating the same miRNA, hence influencing the activity of the same target genes, exemplified by the regulation of hsa-miR-17-5p by E2F1 and NFKB1, subsequently targets MMP2, VEGFA, IL-8, and E2F1. These findings may be explained by temporary, but spatial interactions among mRNAs and miRNAs. TF, via miRNA, may indirectly influence the activity of other genes. This includes REL which regulates hsa-miR-21-5p, which targets STAT3, E2F1, NFKB1, MMP-2 and VEGFA. The TF-DEM network demonstrates that miRNA target TF sets, which physically interact with each other to silence the functional unit. The existence of a direct negative feedback loop between miRNA and TF highlights the limitation of functional units, as illustrated for NF-KB1, the limiting factor in the transcriptional regulation protein complex. It is noteworthy that miRNA and TF have similar regulatory nature, such as pleiotropy, regulation, and network motifs, which yield specific interactions that are propagated throughout the network.

While not abnormally expressed, a number of the network host genes and associated miRNAs appear to be involved in RPL. Accordingly, a single miRNA is depicted to be localized within multiple host genes, exemplified by the presence of hsa-miR-101-3p in RCL1 and within LOC100129106, and the localization of hsa-miR-149-5p in GPC1 and PP14571. This provides for exploring more in depth the possible relationships between host genes and associated miRNA, especially when their miRNAs are differentially expressed.

### Analysis of PPI network of the significantly RPL-associated proteins

**Fig. 3:**
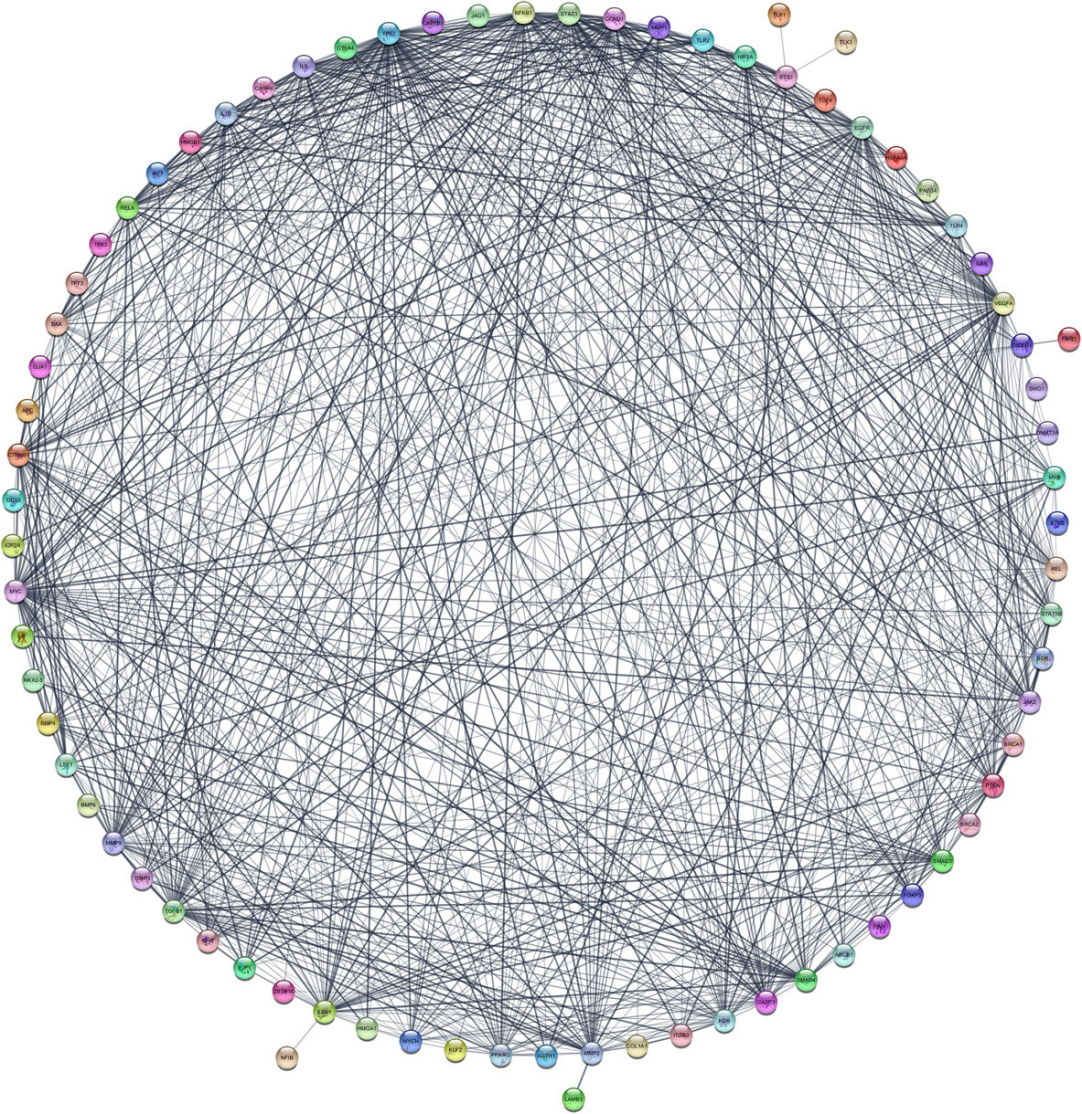
RPL-PPI Network

**Fig. 4:**
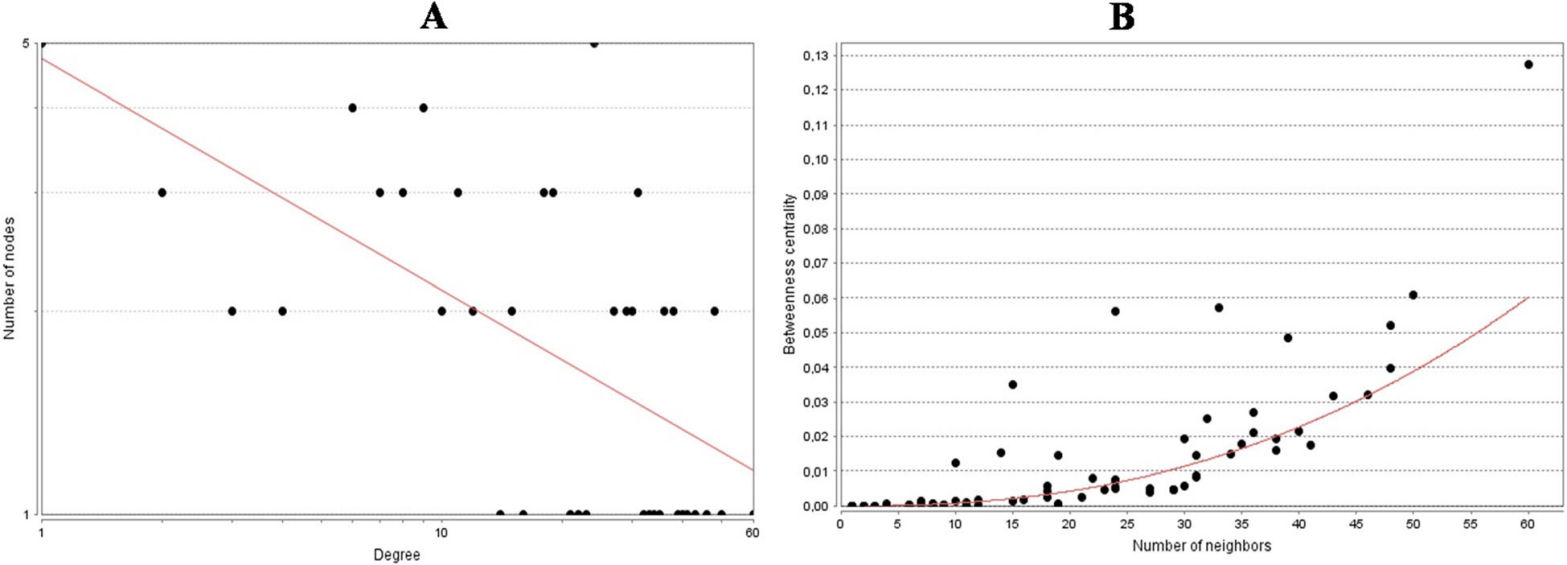
Degree and betweenness Centrality Distribution Curve of the PPI Network of RPL is Illustrated. The red line indicates the power law. (A) Degree distribution: The degree distribution in the scale-free network in logarithmic scale represents the existence of a small number of nodes with high degree (hubs) and a large of nodes with a low degree. (B) Betweenness centrality distribution

**Fig. 5:**
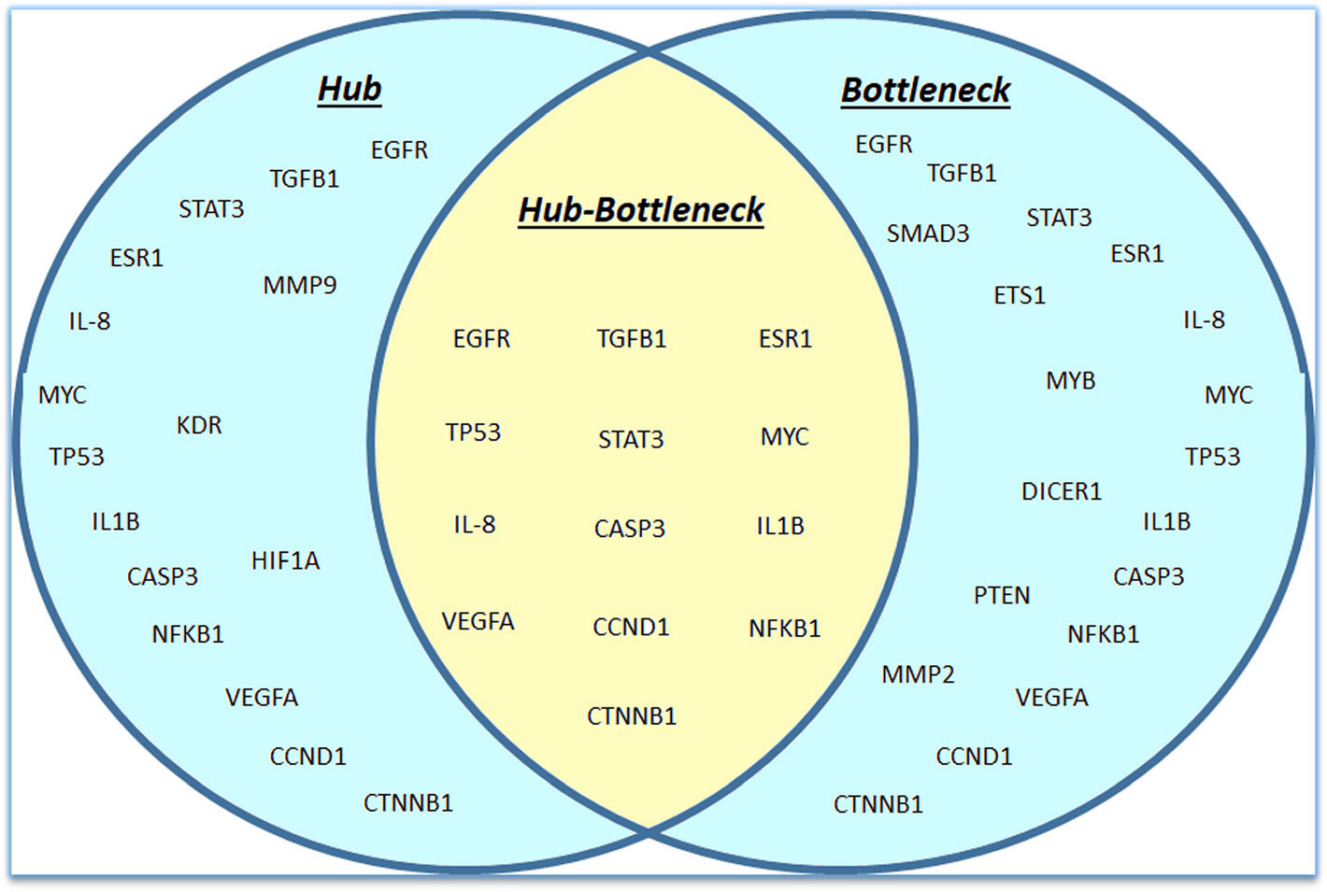
The List of hub-bottleneck proteins. Common proteins (13 proteins), selected in hub and bottleneck states, and represented as hub-bottleneck proteins

PPI networks of the significantly RPL-associated proteins, identified 77 nodes and 763 edges (Fig. 3). In addition, the power law of node degree distribution and centrality distribution were also performed (Fig. 4). Insofar as PPI networks are scale-free networks with power-law degree distribution, most nodes (proteins) have few connections to other node, whereas other nodes (specifically hubs) are connected to other nodes in the network. Common proteins (13 proteins), selected in hub and bottleneck states, and represented as hub-bottleneck proteins (Fig. 5), included EGFR, IL8, TP53, VEGFA, STAT3, TGFB1, CCND1, CTNNB1, CASP3, IL1B, MYC, ESR1 and NF-κB1.

Protein complexes (clusters) were identified by MCODE algorithm, and the global network was partitioned into three clusters, evaluated according to functionality (Table 3); function annotation performed using BiNGO plugin of Cytoscape (Table 4). Cluster 1 focused on regulation of cell proliferation, positive regulation of biological and developmental processes. Cluster 2 proteins were related to negative regulation of striated muscle tissue and muscle organ development. On the other hand, Cluster 3 members dealt with positive regulation of transcription from RNA polymerase II promoter, and DNA-dependent and positive regulation of RNA metabolic process.

**Table 3:** Significant Clusters Related to the PPI Network of RPL and Their Properties

Clusters	Details
	Rank: 1 Nodes: 27 Edges: 308 Cluster Score: 23.692 Seed node: P35222 Seed Protein: CTNNB1 Seed MCODE score: 18.0
	Rank: 2 Nodes: 3 Edges: 3 Cluster Score: 3 Seed node: - Seed Protein: - Seed MCODE score: 9.0
	Rank: 3 Nodes: 7 Edges: 8 Cluster Score: 2.667 Seed node: Q13485 Seed Protein: SMAD4 Seed MCODE score: 13.450

**Table 4:** Functional annotations of the protein complexes (clusters)

GO-ID	corr *p*-value	x	Description
Module 1			
42,127	4.3349E-18	20	regulation of cell proliferation
48,518	6.0837E-18	25	positive regulation of biological process
50,793	1.8202E-17	19	regulation of developmental process
48,522	1.8202E-17	24	positive regulation of cellular process
42,221	2.9470E-17	22	response to chemical stimulus
51,239	6.8692E-17	20	regulation of multicellular organismal process
9893	9.7918E-16	19	positive regulation of metabolic process
51,173	1.0140E-15	17	positive regulation of nitrogen compound metabolic process
42,981	1.1601E-15	18	regulation of apoptosis
43,067	1.2325E-15	18	regulation of programmed cell death
10,941	1.2938E-15	18	regulation of cell death
31,328	1.9038E-15	17	positive regulation of cellular biosynthetic process
9891	2.2668E-15	17	positive regulation of biosynthetic process
10,628	3.7320E-15	16	positive regulation of gene expression
10,604	4.0493E-15	18	positive regulation of macromolecule metabolic process
Module 2			
45,843	2.1117E-4	2	negative regulation of striated muscle tissue development
48,635	2.1117E-4	2	negative regulation of muscle organ development
Module 3			
45,944	5.4874E-7	6	positive regulation of transcription from RNA polymerase II promoter
45,893	9.0278E-7	6	positive regulation of transcription, DNA-dependent
51,254	9.0278E-7	6	positive regulation of RNA metabolic process
45,941	1.4582E-6	6	positive regulation of transcription
10,628	1.5514E-6	6	positive regulation of gene expression
45,935	1.9401E-6	6	positive regulation of nucleobase, nucleoside, nucleotide and nucleic acid metabolic process
10,557	1.9401E-6	6	positive regulation of macromolecule biosynthetic process
51,173	1.9401E-6	6	positive regulation of nitrogen compound metabolic process
31,328	2.3309E-6	6	positive regulation of cellular biosynthetic process
9891	2.3309E-6	6	positive regulation of biosynthetic process
10,552	2.3309E-6	4	positive regulation of gene-specific transcription from RNA polymerase II promoter
6357	2.3309E-6	6	regulation of transcription from RNA polymerase II promoter
43,193	6.8613E-6	4	positive regulation of gene-specific transcription
10,604	7.9390E-6	6	positive regulation of macromolecule metabolic process
31,325	8.6648E-6	6	positive regulation of cellular metabolic process

## Discussion

Bioinformatics analysis was utilized in identifying key miRNAs and genes to RPL. While earlier studies focused mostly on specific genes or metabolic pathways, our study included plethora of data generated to elucidate the role of interacting elements, and to understand their functional contribution in RPL. Our results identified select miRNA and TFs, and mapped their interaction and regulation for experimental validation in RPL. This study is the first to use bioinformatics in identifying and characterizing miRNA and gene expression profile in RPL. It is also the first to analyze the interaction among gene products, and to address the potential functionality of these genes in RPL.

The use of “omics” for identifying genes and effector mechanisms in RPL has challenges, mainly selection of study subjects (female patients, couple with miscarriages, fetus/placenta, and controls). The rely on genetic association studies focusing on candidate genes with pathological effect in RPL has limitations. While polymorphic variants of∼100 genes were investigated for possible association with RPL, results obtained were often inconclusive, and their diagnostic and prognostic utility remains questionable. Future directions in investigating biomolecular risk factors for RPL needs to integrate high-throughput profiling of genomes, transcriptomes, proteomes, metabolomes, and interactomes.

We identified 10 real hub miRNAs, and a further 80 mRNAs to be associated with RPL, and addressed their functionality through identification of miRNA-mRNA signaling pathways contributing to RPL pathogenesis. TGF-β was the most significant pathway enriched by the upregulated miRNAs, namely hsa-miR-155-5p, hsa-miR-16-5p, hsa-miR-92a-3, hsa-miR-145-5p, hsa-miR-221-3p, hsa-miR-101-3p, and hsa-miR-146a-5p. These miRNA target MYC gene, previously shown to be involved in trophoblast proliferation and apoptosis in RPL [35]. The anti-invasive action of TGF-β on trophoblast is mediated by multiple mechanisms, including up-regulation of integrin expression, and reduction in migratory ability of invasive trophoblast [36]. TGF-β also reduces matrix degradation via upregulating MMP tissue inhibitor expression.

Upregulated hsa-miR-16-5p targeted 31 genes in TGF-β signaling, and was involved in > 5 pathways, including adherens junction pathway. Differently expressed miRNAs affecting Wnt signaling, cell cycle, and adhesion molecules, were linked with defective embryo implantation in RPL [37]. Other pathways included MAPK pathway, which contributes to maintenance of normal pregnancy, and alteration in this pathway precipitates RPL [28]. MAPK pathway was influenced by hsa-miR-16-5p and p53 pathways, and modulated by hsa-miR-16-5p and hsa-miR-155-5p. In addition, altered p53 signaling pathway, involved in apoptosis and cell cycle regulation [38], results in enhanced placental apoptosis, and RPL through upregulation of hsa-miR-155-5p [39]. As overexpression of hsa-miR-155-5p enhances, while its antagonism impairs NK cell-mediated cytotoxic activity, this suggesting targeting hsa-miR-155 in managing RPL [40]. Similarly, 15 pathways affected by downregulated miRNAs were noted, including thyroid hormone signaling and prolactin signaling pathways. Altered thyroid hormone level can lead to abnormal sexual development, menstruation irregularity, and likely RPL, while hyperprolactinemia affects hypothalamic-pituitary-ovarian axis, resulting in a shorter luteal phase [41].

Regulation of transcriptional and post-transcriptional events were obtained by constructing curated miRNA-TF regulatory network, the smaller significant sub-network modules centering on NF-κB1 gene was key. Direct TF-miRNA feedback loops identified two NF-κB1 loops involving hsa-miR-155-5p and hsa-miR-146a-5p, and a third involving NF-κB1 and hsa-miR-21-5p. NF-κB1 regulates innate and adaptive immunity, and induces inflammation by stimulating the expression of pro-inflammatory genes, especially IL-6 and IL-8 [42]. Similarly, hsa-miR-146a-5p negatively regulates the expression of IL-6 and IL-8 [43]. Moreover, NF-κB1 activity is regulated by the ubiquitin-protein ligase pellino homolog 1, the expression of which is negatively regulated by hsa-miR-21-5p and thus is inhibitory of NF-κB1 [44]. On the other hand, hsa-miR-21-5p was shown to be activated by IL-6 in a STAT3-dependent manner [45]. A strong link between miR-146a-5p and hsa-miR-21-5p, and with NF-κB1 was seen, predicting that NF-κB1 interaction with hsa-miR-146a-5p affects IL-8 expression, suggesting feedback loops involving NF-κB1 and hsa-miR-146a-5p or hsa-miR-21-5p for controlling RPL-associated inflammation. Earlier studies documented negative feedback comprising hsa-miR-21-5p with NF-κB1, which was linked with altered NF-κB1 and IL-6 activity, and additional negative feedback loop provided by hsa-miR-146 [46]. Accordingly, differences in the strength of the two loops provides for the needed signals for RPL development.

By providing target-specific post-transcriptional repression mechanisms, miRNA-TF network underscores the complexity of TF-miRNA motifs. Compared to TF, miRNA can quickly terminate, or alternatively resume protein translation by binding to, or disassociating from an already transcribed mRNA. This provides for rapid and adaptive changes in gene expression. Although they are subject to differential expression, the exact role of miRNA in RPL remains to be seen. The proposed transcriptional network could explain this involvement. For example, while NF-κB1-hsa-miR-146a-5p loops underscore miRNA effect in sustaining normal immune homeostasis, abnormal hsa-miR-146a-5p expression can drive inflammation; a hypothesis that could not have been made otherwise.

PPI network analysis allows for discrimination of key nodes, and is useful as network biomarker discovery in RPL. Based on their significance in the network, PPI network of 77 RPL-associated proteins were constructed as predictors of drug targets. Crucial nodes which interacted with and controlled the expression of other network nodes, were introduced as essential in RPL PPI network. Localized in Cluster 1, 13 key nodes were found to be related to RPL, individually or in combination, of which CTNNB1 appears the most significant. Mothers against decapentaplegic homolog 4 (SMAD4) protein was also found to be a crucial node in the PPI network, and was also found as a seed protein in Cluster 3. SMAD4, previously reported as key component in TGF-β signaling [47], are intracellular transducers of TGF-β superfamily, and PSG9/SMAD4 complex recruite cytoplasmic SMAD2/3 forming a complex, which enhanced SMAD4 nuclear retention [48]. PSG9-SMAD4 complex was shown to activate the expression of angiogenesis-related genes, including VEGFA, thus contributing to the endometrial angiogenesis affecting both conception and fetal development [49].

STAT3 is a crucial hub-bottleneck protein involved in cytokine and growth factor signaling, and is a key regulator of anti-inflammatory signaling pathway. Altered STAT activity was implicated in adverse pregnancy outcomes, and a 3′-UTR *STAT3* variant contributes to RPL by precipitating a local inflammatory state [50]. Since endometrium signaling involves STAT pathway [50], defective STAT signaling due to attenuated endometrial STAT3-associated tyrosine kinase activity [51, 52], or altered availability of cytokine/growth factor-driven receptor engagement was seen in RPL, suggesting a role in unexplained infertility. CTNNB1, identified as the seed node in Cluster 1, is present in 21 KEGG pathways, including Wnt signaling pathway, suggest that altered CTNNB1 expression contributed to RPL pathogenesis. With regards to apoptosis-related genes and pathways, several evidences confirmed apoptosis of trophoblast cells in early pregnancy [53]. Implantation and growth of blastocyst, regression and reconstruction of decidual tissues, remodeling of placental structure and other processes are closely related to apoptosis, were also present [53]. A balance between apoptosis and proliferation of villous and decidual cells during pregnancy and adverse pregnancy outcome, including RPL, are commonly associated with the excessive apoptotic cells.

## Conclusions

Using bioinformatics, results of the present study provides for theoretical framework toward personalized RPL therapy. Hub-bottleneck proteins, had critical role and high importance in physiopathology of RPL, can serve a diagnostic, even prognostic, role in RPL pathobiology. It is tempting to speculate that several of the identified miRNAs and proteins can potentially serve as diagnostic or therapeutic agents. To this end, prioritization of these targets, and validation of the results using direct cellular analysis, and where possible examination of the impact of the blockade of their expression and activity will be needed for further examination. We favor the notion that drugs designed against these proteins can be important in controlling and managing of RPL.

## Supplementary information

**Additional file 1: Table S1.** Data banks/repositories corresponding to datasets analyzed in this study.

**Additional file 2: Table S2.** Accession Number of Identified miRNA.

## Data Availability

Three types of specific datasets were used. First, experimentally validated datasets of human miRNAs and target genes were collected from miRtarbase (http://mirtarbase.mbc.nctu.edu.tw) available at (http://mirtarbase.mbc.nctu.edu.tw/cache/download/7.0/hsa_MTI.xlsx). The second dataset consisting of targeting associations of human miRNAs and genes was collected from miRecords (http://c1.accurascience.com/miRecords/), which is available at (http://c1.accurascience.com/miRecords/download_data.php?v=4). Lastly, experimentally confirmed TF-miRNA regulations in TransmiR (http://www.cuilab.cn/transmir) was used for analyzing the interplay between TFs and miRNAs, and is available at (http://www.cuilab.cn/files/images/transmir2/download/literature/hsa.xlsx). The names and direct links of the data banks/repositories corresponding to the datasets obtained from web-based sources and subsequently analysed in the study are found in Suplementary Table 1: *Data banks/repositories corresponding to datasets analyzed in this study.* The accession number and direct web links of the identified miRNA appear in Additional file 2: Table S2.

## Abbreviations

BiNGO: Biological Networks Gene Ontology
DEGs: Differentially expressed genes
DEMs: Differentially expressed miRNAs
ESAS: Exome sequencing analysis studies
FDR: False discovery rate
GAD: Genetic Association Database
GWAS: Genome wide association studies
GO: Gene ontology
KEGG: Kyoto Encyclopedia of Genes and Genomes
MCODE: Molecular Complex Detection
miRNAs: microRNAs
MTI: miRNA-target interaction
OMIM: Online Mendelian Inheritance in Man
PPI: Protein-protein interaction
RPL: Recurrent pregnancy loss
STRING: Search Tool for Retrieval of Interacting Genes
TF: Transcription factor
TFBS: Transcription factor binding sites
