# Supplementary material for: Identification of genes and miRNA associated with idiopathic recurrent pregnancy loss: an exploratory data mining study
Source: BMC Med Genomics. 2020 Jun 1;13:75. doi: 10.1186/s12920-020-00730-z (PMC7268288; doi:10.1186/s12920-020-00730-z)
Supplement: Supplementary file 1 — Additional file 1: Table S1. Data banks/repositories corresponding to datasets analyzed in this study. [file 12920_2020_730_MOESM1_ESM.docx]

**Supplementary Table 1**

Data banks/repositories corresponding to datasets analyzed in this study

| **Dataset** | **Full name** | **Direct Web Link** |
| --- | --- | --- |
| BiNGO | Biological Networks Gene Ontology | [www.psb.ugent.be/cbd/papers/BiNGO/](http://www.psb.ugent.be/cbd/papers/BiNGO/) |
| BridgeDb | Identifier mapping framework | <https://bridgedb.github.io/> |
| ChIPBase | Chromatin ImmunoPrecipitation Base | <http://rna.sysu.edu.cn/chipbase/> |
| CyTargetlinker | Cytscape Target Linker | <http://apps.cytoscape.org/apps/cytargetlinker> |
| Cytoscape | Cytoscape | [www.cytoscape.org/](http://www.cytoscape.org/) |
| DIANA-miRPath | DNA Intelligent Analysis | [www.microrna.gr/miRPathv2](http://www.microrna.gr/miRPathv2) |
| GAD | Genetic Association Database | <https://geneticassociationdb.nih.gov/> |
| GeneCards | GeneCards | <https://www.genecards.org/> |
| HMDD | Human microRNA Disease Database | <http://www.cuilab.cn/hmdd> |
| Phenopedia | HuGE Navigator Phenopedia | <https://phgkb.cdc.gov/PHGKB/startPagePhenoPedia.action> |
| KEGG | Kyoto Encyclopedia of Genes and Genomes | <https://www.genome.jp/kegg/kegg3a.html> |
| MCODE | Molecular Complex Detection | [ftp.mshri.on.ca/pub/BIND/Tools/MCODE](ftp://ftp.mshri.on.ca/pub/BIND/Tools/MCODE) |
| miR2Disease | miR2Disease | <http://mir2disease.org/> |
| miRBase | microRNA database | [www.mirbase.org/](http://www.mirbase.org/) |
| OMIM | Online Mendelian Inheritance in Man | <https://omim.org/> |
| Orphanet | Orphanet | <https://www.orpha.net/consor/cgi-bin/index.php> |
| **Dataset** | **Full name** | **Direct link** |
| Orphanet | Orphanet | <https://www.orpha.net/consor/cgi-bin/index.php> |
| P-match | P-match | [www.gene-regulation.com/cgi-bin/pub/programs/pmatch/bin/p-match.cgi](http://www.gene-regulation.com/cgi-bin/pub/programs/pmatch/bin/p-match.cgi). |
| PhenomiR | PhenomiR | <http://mips.helmholtz-muenchen.de/phenomir/> |
| RSAT | Regulatory Sequence Analysis Tools | <http://rsat.sb-roscoff.fr/> |
| STRING | Search Tool for Retrieval of Interacting Genes | [www.stringdb.org/](http://www.stringdb.org/) |
| TRANSFAC^®^ | TRANScription FACtor database | <http://gene-regulation.com/pub/databases.html> |
| miRTarBase | miRTarBase | <http://mirtarbase.mbc.nctu.edu.tw/php/index.php> |
| miRTarBase dataset | miRTarBase dataset | <http://mirtarbase.mbc.nctu.edu.tw/cache/download/7.0/hsa_MTI.xlsx> |
| miRecords | miRecords | <http://c1.accurascience.com/miRecords/> |
| miRecords dataset | miRecords dataset | <http://c1.accurascience.com/miRecords/download_data.php?v=4> |
| TransmiR | TransmiR | <http://www.cuilab.cn/transmir> |
| TransmiR Dataset | TransmiR Dataset | <http://www.cuilab.cn/files/images/transmir2/download/literature/hsa.xlsx> |
