# Supplementary material for: Identification of genes and miRNA associated with idiopathic recurrent pregnancy loss: an exploratory data mining study
Source: BMC Med Genomics. 2020 Jun 1;13:75. doi: 10.1186/s12920-020-00730-z (PMC7268288; doi:10.1186/s12920-020-00730-z)
Supplement: Supplementary file 2 — Additional file 2: Table S2. Accession Number of Identified miRNA. [file 12920_2020_730_MOESM2_ESM.docx]

Supplementary Table 2

**Accession Number of Identified miRNA**

| **Accession Number ^1^** | **Name** | **Direct Link** |
| --- | --- | --- |
| [MIMAT0000076](http://www.mirbase.org/cgi-bin/query.pl?terms=MIMAT0000076) | hsa-miR-21-5p | http://www.mirbase.org/cgi-bin/mature.pl?mature_acc=MIMAT0000076 |
| [MIMAT0000646](http://www.mirbase.org/cgi-bin/query.pl?terms=MIMAT0000646) | hsa-miR-155-5p | http://www.mirbase.org/cgi-bin/mature.pl?mature_acc=MIMAT0000646 |
| [MIMAT0000069](http://www.mirbase.org/cgi-bin/query.pl?terms=MIMAT0000069) | hsa-miR-16-5p | http://www.mirbase.org/cgi-bin/mature.pl?mature_acc=MIMAT0000069 |
| [MIMAT0000070](http://www.mirbase.org/cgi-bin/query.pl?terms=MIMAT0000070) | hsa-miR-17-5p | http://www.mirbase.org/cgi-bin/mature.pl?mature_acc=MIMAT0000070 |
| [MIMAT0000449](http://www.mirbase.org/cgi-bin/query.pl?terms=MIMAT0000449) | hsa-miR-146a-5p | http://www.mirbase.org/cgi-bin/mature.pl?mature_acc=MIMAT0000449 |
| [MIMAT0000092](http://www.mirbase.org/cgi-bin/query.pl?terms=MIMAT0000092) | hsa-miR-92a-3p | http://www.mirbase.org/cgi-bin/mature.pl?mature_acc=MIMAT0000092 |
| [MIMAT0000437](http://www.mirbase.org/cgi-bin/query.pl?terms=MIMAT0000437) | hsa-miR-145-5p | http://www.mirbase.org/cgi-bin/mature.pl?mature_acc=MIMAT0000437 |
| [MIMAT0000074](http://www.mirbase.org/cgi-bin/query.pl?terms=MIMAT0000074) | hsa-miR-19b-3p | http://www.mirbase.org/cgi-bin/mature.pl?mature_acc=MIMAT0000074 |
| [MIMAT0000278](http://mirbase.org/cgi-bin/mature.pl?mature_acc=MIMAT0000278) | hsa-miR-221-3p | http://www.mirbase.org/cgi-bin/mature.pl?mature_acc=MIMAT0000278 |
| [MIMAT0000099](http://www.mirbase.org/cgi-bin/query.pl?terms=MIMAT0000099) | hsa-miR-101-3p | http://www.mirbase.org/cgi-bin/mature.pl?mature_acc=MIMAT0000099 |
| [MIMAT0002809](http://www.mirbase.org/cgi-bin/mature.pl?mature_acc=MIMAT0002809) | hsa-miR-146b-5p | http://www.mirbase.org/cgi-bin/mature.pl?mature_acc=MIMAT0002809 |
| [MIMAT0000443](http://www.mirbase.org/cgi-bin/mature.pl?mature_acc=MIMAT0000443) | hsa-miR-125a-5p | http://www.mirbase.org/cgi-bin/mirna_entry.pl?acc=MIMAT0000443 |
| [MIMAT0005792](http://www.mirbase.org/cgi-bin/mature.pl?acc=MIMAT0005792) | hsa-miR-320b | http://www.mirbase.org/cgi-bin/mature.pl?acc=MIMAT0005792 |
| [MIMAT0026478](http://www.mirbase.org/cgi-bin/mature.pl?mature_acc=MIMAT0026478) | hsa-miR-133a-3p | http://www.mirbase.org/cgi-bin/mirna_entry.pl?acc=MIMAT0000427 |
| [MIMAT0004570](http://www.mirbase.org/cgi-bin/mature.pl?mature_acc=MIMAT0004570) | hsa-miR-223-3p | http://www.mirbase.org/cgi-bin/mirna_entry.pl?acc=MIMAT0000280 |
| [MIMAT0004699](http://www.mirbase.org/cgi-bin/mature.pl?mature_acc=MIMAT0004699) | hsa-miR-148b-3p | http://www.mirbase.org/cgi-bin/mirna_entry.pl?acc=MIMAT0000759 |
| [MIMAT0000245](http://www.mirbase.org/cgi-bin/mature.pl?mature_acc=MIMAT0000245) | hsa-miR-30d-5p | http://www.mirbase.org/cgi-bin/mirna_entry.pl?acc=MIMAT0000245 |
| [MIMAT0004587](http://www.mirbase.org/cgi-bin/mature.pl?mature_acc=MIMAT0004587) | hsa-miR-23b-3p | http://www.mirbase.org/cgi-bin/mirna_entry.pl?acc=MIMAT0000418 |
| [MIMAT0004748](http://www.mirbase.org/cgi-bin/mature.pl?mature_acc=MIMAT0004748) | hsa-miR-423-3p | http://www.mirbase.org/cgi-bin/mirna_entry.pl?acc=MIMAT0001340 |
| [MIMAT0000256](http://www.mirbase.org/cgi-bin/mature.pl?mature_acc=MIMAT0000256) | hsa-miR-181a-5p | http://www.mirbase.org/cgi-bin/mirna_entry.pl?acc=MIMAT0000256 |
| [MIMAT0001341](http://www.mirbase.org/cgi-bin/mature.pl?mature_acc=MIMAT0001341) | hsa-miR-424-5p | http://www.mirbase.org/cgi-bin/mature.pl?mature_acc=MIMAT0001341 |
| [MIMAT0000245](http://www.mirbase.org/cgi-bin/mature.pl?mature_acc=MIMAT0000245) | hsa-miR-30d-5p | http://www.mirbase.org/cgi-bin/mirna_entry.pl?acc=MIMAT0000245 |
| [MIMAT0000435](http://www.mirbase.org/cgi-bin/mature.pl?mature_acc=MIMAT0000435) | hsa-miR-143-3p | http://www.mirbase.org/cgi-bin/mirna_entry.pl?acc=MIMAT0000435 |
| [MIMAT0000419](http://www.mirbase.org/cgi-bin/mature.pl?mature_acc=MIMAT0000419) | hsa-miR-27b-3p | http://www.mirbase.org/cgi-bin/mirna_entry.pl?acc=MIMAT0000419 |
| [MIMAT0003223](http://www.mirbase.org/cgi-bin/mature.pl?mature_acc=MIMAT0003223) | hsa-miR-559 | http://www.mirbase.org/cgi-bin/mirna_entry.pl?acc=MI0003565 |
| **Accession Number ^1^** | **Name** | **Direct Link** |
| [MIMAT0000710](http://www.mirbase.org/cgi-bin/mature.pl?mature_acc=MIMAT0000710) | hsa-miR-365a-3p | http://www.mirbase.org/cgi-bin/mirna_entry.pl?acc=MIMAT0000710 |
| [MIMAT0005827](http://www.mirbase.org/cgi-bin/mature.pl?mature_acc=MIMAT0005827) | hsa-miR-1182 | http://www.mirbase.org/cgi-bin/mirna_entry.pl?acc=MI0006275 |
| [MIMAT0016915](http://www.mirbase.org/cgi-bin/mature.pl?mature_acc=MIMAT0016915) | hsa-miR-4284 | http://www.mirbase.org/cgi-bin/mirna_entry.pl?acc=MI0015893 |
| [MIMAT0016899](http://www.mirbase.org/cgi-bin/mature.pl?mature_acc=MIMAT0016899) | hsa-miR-4264 | http://www.mirbase.org/cgi-bin/mirna_entry.pl?acc=MI0015877 |
| [MIMAT0019234](http://www.mirbase.org/cgi-bin/mature.pl?mature_acc=MIMAT0019234) | hsa-miR-4474-5p | http://www.mirbase.org/cgi-bin/mirna_entry.pl?acc=MIMAT0019234 |
| [MIMAT0004495](http://www.mirbase.org/cgi-bin/mature.pl?mature_acc=MIMAT0004495) | hsa-miR-22-5p | http://www.mirbase.org/cgi-bin/mirna_entry.pl?acc=MIMAT0004495 |
| [MIMAT0026484](http://www.mirbase.org/cgi-bin/mature.pl?mature_acc=MIMAT0026484) | hsa-miR-372-5p | http://www.mirbase.org/cgi-bin/mirna_entry.pl?acc=MIMAT0026484 |
| [MIMAT0001631](http://www.mirbase.org/cgi-bin/mature.pl?mature_acc=MIMAT0001631) | hsa-miR-451a | http://www.mirbase.org/cgi-bin/mirna_entry.pl?acc=MI0001729 |
| [MIMAT0000450](http://www.mirbase.org/cgi-bin/mature.pl?mature_acc=MIMAT0000450) | hsa-miR-149-5p | http://www.mirbase.org/cgi-bin/mirna_entry.pl?acc=MIMAT0000450 |
| [MIMAT0000257](http://www.mirbase.org/cgi-bin/mature.pl?mature_acc=MIMAT0000257) | hsa-miR-181b-5p | http://www.mirbase.org/cgi-bin/mirna_entry.pl?acc=MIMAT0000257 |

1. www.mirbase.org
